# The Role of *Areca catechu* Extract on Decreasing Viscosity of Vegetable Oils

**DOI:** 10.1155/2021/8827427

**Published:** 2021-07-29

**Authors:** Wardoyo, Agung S. Widodo, Widya Wijayanti, I. N. G. Wardana

**Affiliations:** ^1^Department of Mechanical Engineering, Faculty of Engineering, Brawijaya University, Malang 65145, Indonesia; ^2^Department of Mechanical Engineering, Faculty of Engineering, State University of Jakarta, Jakarta 13220, Indonesia

## Abstract

The direct use of vegetable oil is constrained by vegetable oils' viscosity, which is relatively high. Therefore, this study discusses reducing the vegetable oils viscosity molecularly, using natural additives of *Areca* extract. The role of *Areca* extract on diminishing the viscosity of vegetable oil was simulated utilizing HyperChem software. Then, the viscosity was verified using the ASTM D445 method. The results show that the epicatechin in the *Areca* extract generates a local magnetic field on its aromatic ring that plays the role of energizing electron mobility in vegetable oil. On the one hand, increased electron mobility decreases oil polarity but, on the other hand, increases the London force via a temporary dipole charge. Simultaneously, it relaxes oil molecules that tend to increase molecule distance decreasing the London force. Since viscosity is proportional to the London force and oil polarity; thus, these three phenomena determine the role of *Areca* extract on the oil viscosity. The viscosity reduction is smaller at a larger number of the double bonds in the fatty acid chain since the local magnetic field's induction on the electron spin is interrupted in the double bonds

## 1. Introduction

The need for vegetable oil to serve as an alternative energy source has increased as the Earth's supply of fossil energy sources has been depleted [1]. According to BP's 2018 Statistical Review of World Energy report, global oil reserves stood at 239,300 million tons, while the global oil consumption rate was 4,621.9 million tons per year. If these conditions are maintained, the world's oil reserves could be exhausted by 2070 [2]. One solution to this problem is the use of vegetable oil as an energy source. Vegetable oil is abundantly available in its natural form from various sources, which can be quickly renewed and are readily biodegradable [3].

However, the direct use of crude vegetable oil is limited by its high viscosity. The high viscosity results in nonideal atomization, a low evaporation rate, and incomplete combustion, and it also shortens the life of the fuel filter [4]. Additionally, high-viscosity fuels are known to shorten the lifetimes of other fuel system components [5].

Vegetable oil is composed of glycerol and fatty acid molecules. It mostly comprises triglyceride molecules as well as a small portion of diglycerides and monoglycerides [6, 7]. Vegetable oil can be converted into biodiesel by separating the glycerol from the fatty acids. Biodiesel is produced by means of degumming, esterification, trans-esterification, hydrogenation, and catalytic-cracking processes [8]. All of these processes require enormous costs when converting vegetable oil into biodiesel; thus, biodiesel's price is 10% to 50% more expensive than diesel fuel [9].

Many researchers have sought to identify methods of reducing the cost of producing biodiesel. These methods include attempts to find alternative means of reducing vegetable oil viscosity, for example, using preheating, mixing, and microemulsion methods [10–13]. The mixing method is among the methods suggested in prior studies to be effective, inexpensive, and fast in relation to reducing the viscosity of vegetable oil. Over the past decade, studies concerning the mixing of vegetable oil-diesel fuel, vegetable oil-alcohol fuel, and vegetable oil-alcohol-diesel fuel have shown that vegetable oil's viscosity drops in proportion to that of diesel fuel. Vegetable oil-diesel fuel blends with different percentage mixtures have been extensively examined by many researchers, with similar results being obtained [14, 15]. Singh et al. [16] reported that a mixture up to a maximum ratio of 20% vegetable oil and 80% diesel fuel could be used directly in diesel engines without any required modifications. However, this mixture still has fossil fuel as its main constituent [16, 17].

Another research branch in this field involves identifying substitutes for diesel fuel by using a vegetable oil-alcohol mixture. Some mixtures used ethanol and methanol as the alcohol components. However, the level of solubility of vegetable oil in these alcohol types was found to be very poor. Therefore, surfactants have been used as additives to improve both the mixing ability and the stability of the mixture [18]. Other researchers have used butanol and pentanol as alcohol components. They have properties closer to diesel fuel and exhibit better solubility and stability than ethanol and methanol. Butanol and pentanol are both low-viscosity alcohols, which can help to reduce the high-viscosity effect of vegetable oil in the mixture [19].

Some previous studies have examined the outcomes of mixing low-viscosity additives (e.g., diesel, methanol, and butanol) with high-viscosity vegetable oil. In brief, the viscosity of such mixtures leads to a new equilibrium value that is lower than the viscosity of the original vegetable oil [3–5, 10]. However, none of these studies have provided an in-depth scientific explanation for the observed phenomenon. Thus, to better understand it, more fundamental studies are needed at the molecular level.

*Areca* nuts mostly contain alkaloids, sugars, lipids, and polyphenols [20]. The chemical structure of *Areca* extract is comprised of polyphenol oligomers, which feature several aromatic rings and hydroxyl groups. The aromatic rings have delocalized electrons that produce small-scale magnetic fields [21], which render them capable of inducing an electron spin in surrounding molecules. Yet, the hydroxyl groups can form hydrogen bonds with the polar head groups found on the lipids [22].

The present study discusses the process behind reducing the viscosity of vegetable oil by utilizing natural additives obtained from *Areca* extract at the molecular level. The magnetic fields associated with the additives energize the spinning electrons in the molecules of the vegetable oil, which causes both their dipole moment and the oil's polarity to decrease. Moreover, the energetic electron motion generates a temporary dipole charge that alters the London force between the oil molecules, thereby increasing the viscosity. At the same time, the active electron relaxes the molecule, which results in decreased viscosity. These three factors—oil polarity, London forces, and relaxed molecule—play essential roles in determining vegetable oil thickness mixed with natural additives.

In terms of the research material, the following types of vegetable oils were chosen: coconut oil (CCO), which is composed of short-chain, saturated, and polar fatty acids; refined palm oil (RPO), which is composed of long-chain, unsaturated, and nonpolar fatty acids; and refined corn oil (RCO), which is composed of long-chain, polyunsaturated, and polar fatty acids. The choice of these three types of oils means that discussing the roles played by the natural additives found in *Areca* extract can be more comprehensive.

## 2. Materials and Methods

### 2.1. Materials

Matured *Areca* fruit was harvested from the *Areca* nut plantations in the Eastern Tanjung Jabung district, Jambi province, Indonesia. The methanol solvent analyst was from Indonesia's smart lab. RPO and RCO were both processed in factories that are usually used for food processing. The CCO was produced by the heating method. The composition of the three vegetable oils is presented in Table 1, completed with the number of carbon atoms (Cn) and double bonds (db) in the fatty acid.

### 2.2. Extraction Methods and Characterization of *Areca* Extract

**Figure 1:**
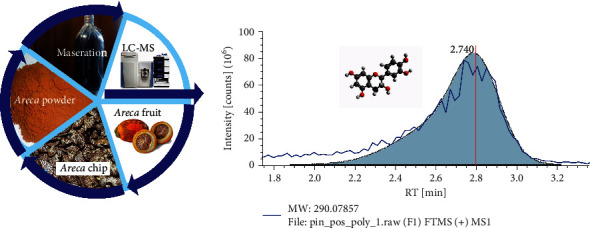
*Areca* extraction process and composition test.

Ripe *Areca* seed was thinly sliced into chips and dried under the sunshine for three days. The dry *Areca* chips were grinded and sieved with a 200-mesh filter. After that, *Areca* powder was extracted by the maceration method using a methanol analyzer solvent. The composition of *Areca* extract was tested using LC-MS-MS metabolomic. The sequence of extraction mechanisms up to the *Areca* nut composition test is shown in Figure 1.

*Areca* nut extract was dissolved in 0.1% formic acid in acetonitrile LC-MS gade, flowing in an analytical column Hypersil GOLD aQ, 50 × 1 mm, particle size 1.9 *μ*m. Analytical flow rate was 40 *μ*L/min with run time of 70 minutes and oven temperature of 30°C. Samples were analyzed on Thermo Scientific Q Exactive High-Resolution Mass Spectrometer, full scan at 70,000 resolution, MS2 dependent data at 17,500 resolution, and run time of 70 minutes. Data was processed using software: Compound Discoverer with mzCloud MS/MS Library.

The LC-MS test result shows that *Areca* extract contains guvacoline, arecoline, choline, epicatechin, trigonelline, and other molecules in small amounts. Epicatechin is among these molecules that can bind to vegetable oils, as shown in Figure 1. Epicatechin has positively charged hydroxyl, which attracts negatively charged carbonyl in vegetable oils. This attractive force makes epicatechin evenly scattered in vegetable oils.

### 2.3. Molecular Dynamics Simulation

**Figure 2:**
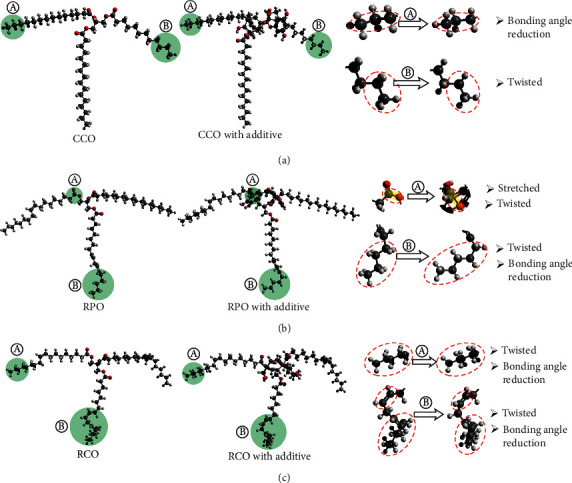
The effect of additives on molecular geometry of vegetable oils. Molecular exchange of (a) CCO, (b) RPO, and (c) RCO.

Simplified dynamic molecular simulation involves only one molecule of epicatechin and one triglyceride molecule, as shown in Figure 2. The dipole moment and electron mobility were obtained using a semiempirical approach using the CNDO calculation of the Polak–Ribiere's conjugate gradient method. The simulation was carried out on convergence limit of 0.01, iteration limit of 50, heat time 0.1 ps, run time of 0.3 ps, time step of 0.0005 ps, starting temperature of 100 K, simulation temperature of 300 K, and temperature step of 30 K.

### 2.4. Mixing Vegetable Oil with *Areca* Extract

The *Areca* extract was added to vegetable oil at doses of 0 ppm, 250 ppm, 500 ppm, 750 ppm, 1000 ppm, and 1250 ppm. Furthermore, the vegetable oil with additive was stirred using magnetic steering at 500 rpm at 50°C for 60 minutes. Then, it was deposited for 12 hours to settle the molecules that cannot mix properly. The test sample used in this study was vegetable oil with an additive that had been mixed stably. Furthermore, the viscosity of vegetable oils with additives was measured using the ASTM D 445 method.

## 3. Results and Discussion

### 3.1. Result

**Figure 3:**
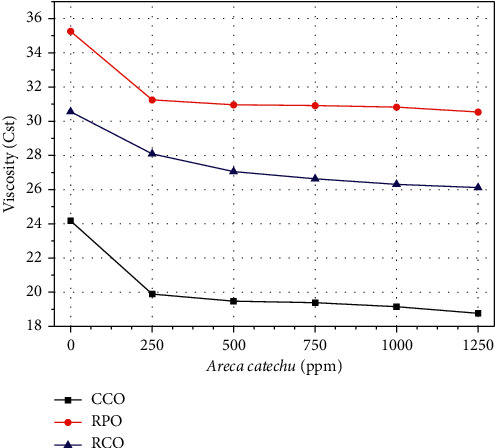
The effect of additives on the kinematic viscosity of vegetable oils.

The viscosity of vegetable oils is affected by the intermolecular attractions of the van der Waals forces. As shown in Table 1, CCO is composed of mostly lauric, myristic, and caprylic acids, which are saturated, short fatty acids, with small molecular masses. The carbonyl's polarity in the fatty acid head is more dominant than its molecular mass, so the CCO is classified into polar fatty acids [24]. The shorter fatty acids in CCO have smaller London forces so that CCO has a lower viscosity than RPO and RCO, as shown in Figure 3. The main compositions of RPO and RCO are palmitic, oleic, and linoleic acids. Palmitic and oleic acids dominate the RPO. Oleic acid is a long single bond carbon chain saturated fatty acid. The longer chains, or larger masses, reduce the polarity of RPO's fatty acid heads and tend to become nonpolar vegetable oils [24]. On the other hand, RCO consists mainly of linoleic acid, long fatty acids, and polyunsaturated acids. The double bonds in linoleic acids turn the molecular geometry into irregular form, making it challenging to transfer momentum. As a result, the viscosity of the RCO is smaller than the RPO, as presented in Figure 3.

### 3.2. Effect of Additives on the Dipole Moments

The dipole moment of vegetable oil is influenced by polarity and molecular mass. The shorter fatty acids and lighter molecular masses dominate CCO, resulting in CCO with highly polar oil with the most significant dipole moments. In contrast, RPO and RCO are long chains with double bond fatty acids having large molecular masses. A double bond has a shorter distance between atoms and more electrons than a single bond. Therefore, electrons' density around the double bond is denser than other regions along the fatty acid chain. RPO and RCO have almost the same molecular mass. However, RCO has more polyunsaturated fatty acids, which makes the electric poles stronger. Hence, RCO's dipole moment is more extensive than RPO.

**Figure 4:**
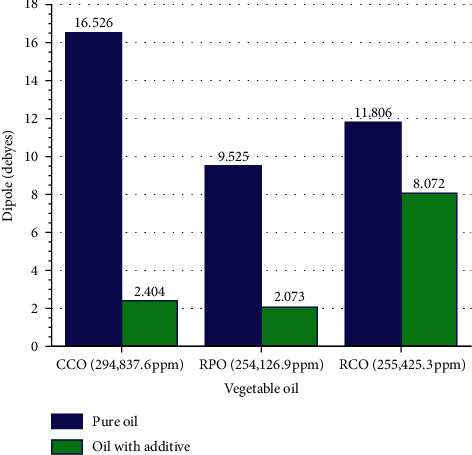
Effect of additives on the dipole moments of vegetable oil.

Figure 4 shows that CCO has a large dipole moment followed by RCO, which is smaller, and RPO is the smallest. The additive magnetic force acts on the entire electron spins along the fatty acid chain from the polar head until the chain's tail. For short fatty acids, the magnetic force induction on the electron spin becomes energetic so that the electron becomes freer to move. Consequently, the dipole moment drops considerably in CCO. A double bond in the fatty acid chain impedes the magnetic induction in the electron spin. As a result, the dipole moment in RCO decreases slightly, whereas that in RPO decreases more but still less than CCO because RCO is composed mostly of polyunsaturated fatty acids. In contrast, RPO is mainly composed of monounsaturated fatty acids.

### 3.3. Effect of Additives on Electron Mobility

**Figure 5:**
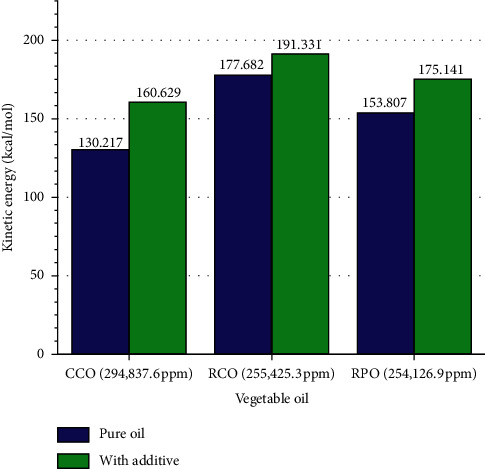
Effect of additives on electron mobility of vegetable.

Electrons always move around the molecule. The movement of an electron's velocity depends on the geometry and polarity of the molecule. The polar electric charge on the carboxyl head attracts the electron tightly in the molecule, so they are less free to move. Electrons are free to move in the larger molecules and the croak fatty acids. The electrons' movement affects the kinetic energy of vegetable oils. The kinetic energy of molecules is higher when the electron is free to move. Linoleic acid dominates RCO. The double bond in linoleic fatty acid hinders the attraction of carboxyl heads electric pole on electrons so that electron mobility increases between the double bonds and the tail of the fatty acid chains [25]. As shown in Figure 5, many double bonds in RCO cause it to have the highest kinetic energy. A few double bonds in the RPO hamper the carboxyl polar attraction on the electron movement so that the kinetic energy of RPO is lower than RCO. In the case of CCO, electron mobility is hindered by significant molecular polarity, so it has the lowest kinetic energy (Figure 5).

With additives, the kinetic energy of vegetable oil increases, as shown in Figure 5. The induction of delocalized electrons in the aromatic ring of additive generates a local magnetic field that energizes the electron spin in vegetable oil—the kinetic energy of the vegetable oil increases. As presented in Figure 5, electron mobility's additive effect is most significant in CCO since it has short and straight chains. For that reason, the induction of the local magnetic field due to delocalized electron evenly spreads along the fatty acid chain. Besides, RPO and RCO have double bonds causing induction of local magnetic field on the electron spin to be agitated so that it cannot be distributed evenly along the chain. RCO has more double bonds than RPO. So, the lowest increasing kinetic energy occurred on RCO, then RPO, and CCO.

### 3.4. Effect of Additives on Viscosity

CCO is more polar than RCO and RPO since it consists of more shorter and straight fatty acids lauric and myristic fatty acids (Table 1). In these fatty acids, the electron is less mobile so that the electric pole in the carboxyl head become strong. On the other hand, RPO consists of 42.31% palmitic acid and 40.90% oleic acid, whereas RCO consists of 57.74% linoleic acid and 27.23% oleic acid. These fatty acids in RPO and RCO are long chain and unsaturated. In those, the electron is more mobile so that the polarity in the carboxyl head of RPO and RCO is weaker than CCO [25]. However, linoleic which is dominant in RCO is a polyunsaturated fatty acid which is slightly more polar than monounsaturated acids in RPO as seen in Figure 4. The polarity of the vegetable oil affects the solubility of the additive. The stronger polarity of vegetable oils attracts stronger additives. So, additives are most easily absorbed in CCO, RCO, and then RPO. At the additive concentration of 1250 ppm, the most significant decrease in viscosity occurred in CCO (28.80%), then RCO (16.99%), and finally RPO (15.44%), as shown in Figure 3.

Phi electron resonance in the epicatechin aromatic ring produces a low-intensity magnetic field [21]. The magnetic field in epicatechin strengthens the electron spin in fatty acid molecules that increases electron mobility. Consequently, the molecule relaxes, stretches, and twists so that the distance between molecules extends. Consequently, the transfer of momentum between molecules reduces and weakens the London force. However, a stronger magnetic field due to the increase in additives attracts stronger molecules and denser. Then, it improves the transfer of momentum and the London force. This result is in line with the findings of Fang et al. [26], and molecular size expands in a low magnetic field and shrinks in a high magnetic field. This phenomenon causes vegetable oil viscosity to drop drastically in the additive concentration of 250 ppm and almost unchanged in the subsequent addition of an additive, as shown in Figure 3. The local magnetic fields have a more significant effect on shorter alkane molecules. Their impact is weakened on longer alkanes with double bonds in carbon chains.

### 3.5. Effect of Additives on Molecular Geometry

The RPO is composed of 51.25% double bonds, with 10.35% polyunsaturated fatty acids, causing the molecules to become curly. While the RCO has 85.61% of the double bond fatty acids in which the 58.38% of it is polyunsaturated, RCO molecules turn into curly and irregular form. In the case of CCO, which is dominated by short saturated fatty acids, molecules' geometry is straighter and neater than other oils.

Induction of the magnetic field generated by electron delocalization pushes electrons away from their orbits. As a result, the bonds between the atoms are stretched and relaxed. The relaxation of fatty acids changes the molecular geometry through twisted and bonding angle reduction along the tail's fatty acid chain. The magnetic force is interrupted by a double bond, so the effect at the end of the fatty acids becomes small. The most significant reduction in the twisted and bonding angle occurs in allylic, the weakest component of vegetable oil. RCO has more allylic than RPO, causing the tail of RCO to be more deformed than other oil (shown in Figure 2).

### 3.6. Discussion

Three intermolecular forces determine the level of viscosity in vegetable oil. First, the dipole moment of vegetable oil shows the quality of polarization. High electron polarization tends to strengthen the bonding energy between molecules and inhibit electron mobility. Second, high electron mobility increases molecule kinetic energy. High electron mobility improves momentum transfer between instantaneous dipole and induction dipole from two different molecules. This condition is called the London force. Finally, large momentum transfers strengthen bonding energy between molecules. Neat molecular shapes increase the transfer of momentum between molecules.

*Areca* extract as an additive has succeeded in reducing vegetable oils' viscosity by decreasing the dipole moment and weakening the transfer of momentum between molecules. The findings of Fang et al. [26] indicate that viscosity is affected by momentum exchange and intermolecular forces. First, *Areca* extract containing epicatechin with delocalizes electrons in its aromatic ring, producing magnetic fields that induce the spin of electrons in their orbitals, resulting in increased mobility of electrons in vegetable oils. Next, the increased electron mobility causes the electron to move away from the center of orbitals, causing the dipole moment to be weakened. Its kinetic energy demonstrates the electron's mobility in vegetable oils. High kinetic energy increases the intensity of bond formation between the temporary dipole and the induction dipole, accelerating the London force.

Additionally, additives cause triglyceride molecules' geometry to become increasingly curly, making them challenging to arrange neatly. The irregular shape causes the distance between molecules to increase, curbing the intensity of electron collisions between molecules. The reduction of transfer momentum between molecules inhibits the progression of the London force. The negligible increase of the London force, combined with the considerable decrease in the dipole moment, causes the total van der Waals energy to contract, reducing the viscosity.

## 4. Conclusions

Epicatechins in *Areca* nut extract have hydroxyl functional groups, and electrons are delocalized on the aromatic ring. The delocalized electrons generate a magnetic field that energizes the electron spins, increasing the electron's mobility. In addition, the magnetic field induction in vegetable oil molecules causes them to change into irregular geometric shapes, widens the distance between molecules, and reduces the frequency of momentum transfer. As a result, the London style is reduced. The hydroxyl group is absorbed in polar vegetable oils and decreases at low molecular polarity. The most significant decrease in viscosity occurred at the additive concentration of 250 ppm. The viscosity of CCO decreased by 21.53%, RPO 12.82%, and RCO 8.80. The intensity of viscosity reduction decreased with the addition of additives, respectively, from concentrations of 500 ppm to 1250 ppm, which were 3.83%, 1.60%, 1.22%, and 0.71%. Thus, *Areca* nut extract as a natural additive has succeeded in reducing the viscosity of vegetable oil with an optimum concentration of 250 ppm. Though lower viscosity could be reached above 1250 ppm, the combustion performance may be lowering which needs to be observed in the next experimental study.

## Figures and Tables

**Table 1:** Fatty acid composition molecules.

Chemical composition	Cn; db	Composition (%)			Molecular mass, g/mole
		CCO [23]	RPO [23]	RCO [23]	
Caprylic acid	8; 0	6.44	0.08	—	144.2114
Capric acid	10; 0	5.62	0.06	—	172.2646
Lauric acid	12; 0	46.70	0.36	—	200.3178
Myristic acid	14; 0	18.75	1.13	—	228.3709
Palmitic acid	16; 0	9.73	42.31	11.88	256.4241
Palmitoleic acid	16; 1	0.11	0.17	0.13	254.4082
Stearic acid	18; 0	2.78	4.27	2.10	284.4772
Oleic acid	18; 1	6.86	40.90	27.23	282.4614
Linoleic acid	18; 2	2.25	10.07	57.74	280.4455
Linolenic acid	18; 3	0.04	0.28	0.64	278.4296
Arachidic acid	20; 0	0.10	0.31	0.32	312.5304
Gondoic acid	20; 1	0.03	0.16	0.35	310.5145

## Data Availability

The data used to support the findings of this study are available from the corresponding author upon request.

## References

[1] Qubeissi M. A., Sazhin S. S., Elwardany A. E. (2017). Modelling of blended diesel and biodiesel fuel droplet heating and evaporation. *Fuel*.

[2] Dudley B.

[3] Ali O. M., Mamat R., Abdullah N. R., Adam Abdullah A. (2016). Analysis of blended fuel properties and engine performance with palm biodiesel e diesel blended fuel. *Renew Energy*.

[4] Yilmaz N., Vigil F. M. (2014). Potential use of a blend of diesel, biodiesel, alcohols and vegetable oil in compression ignition. *Engines*.

[5] Che S., Idroas M. Y., Teoh Y. H., Hamid M. F. (2019). Optimisation of viscosity and density of re fi ned palm oil-melaleuca cajuputi oil binary blends using mixture design method. *Renewable Energy*.

[6] Agarwal D., Agarwal A. K. (2007). Performance and emissions characteristics of jatropha oil (preheated and blends) in a direct injection compression ignition engine. *Applied Thermal Engineering*.

[7] Wahyudi W. I. N. G., Widodo A. S., Wijayanti W. (2018). Improving vegetable oil properties by transforming fatty acid chain length in jatropha oil and coconut. *Energies*.

[8] Zhang M., Wu H. (2015). Effect of major impurities in crude glycerol on solubility and properties of glycerol/methanol/bio-oil blends. *Fuel*.

[9] Leung D. Y. C., Guo Y. (2006). Transesterification of neat and used frying oil. *Optimization for Biodiesel Production*.

[10] Atmanli A., Ileri E., Yuksel B., Yilmaz N. (2015). Extensive analyses of diesel—vegetable oil—*n* -butanol ternary blends in a diesel engine. *Applied Energy*.

[11] Nwafor O. M. I. (2004). Emission characteristics of diesel engine running on vegetable oil with elevated fuel inlet temperature. *Biomass and Bioenergy*.

[12] Bari S., Lim T. H., Yu C. W. (2002). Effects of preheating of crude palm oil (CPO) on injection system. *Performance and Emission of a Diesel Engine*.

[13] Qi D. H., Bae C., Feng Y. M., Jia C. C., Bian Y. Z. (2013). Combustion and emission characteristics of a direct injection compression ignition engine using rapeseed oil based micro-emulsions. *Fuel*.

[14] Agarwal A. K., Dhar A. (2013). Experimental investigations of performance, emission and combustion characteristics of karanja oil blends fuelled. *DICI Engine*.

[15] Fontaras G., Kousoulidou M., Karavalakis G., Bakeas E., Samaras Z. (2011). Impact of straight vegetable oil e diesel blends application on vehicle regulated and non-regulated emissions over legislated and real world driving cycles. *Biomass and Bioenergy*.

[16] Singh P., Varun, Chauhan S. R., Kumar N. (2016). A review on methodology for complete elimination of diesel from CI engines using mixed feedstock. *Renew Sustain Energy Rev*.

[17] Ramadhas A. S. Ã., Jayaraj S., Muraleedharan C. (2004). Use of vegetable oils as I.C. engine fuels—a review. *Renewable Energy*.

[18] Bhimani S., Alvarado J. L., Annamalai K., Marsh C. (2013). Emission characteristics of methanol-in-canola oil emulsions in a combustion chamber. *Fuel*.

[19] Laza T., Bereczky Á. (2011). Basic fuel properties of rapeseed oil-higher alcohols blends. *Fuel*.

[20] Wetwitayaklung P., Phaechamud T. (2006). The study of antioxidant capacity in various parts of *Areca catechu* L.. *Naresuan University Journal: Science and Technology*.

[21] Ajami D., Oeckler O., Simon A., Herges R. (2018). Synthesis of a möbius aromatic hydrocarbon. *Nature*.

[22] Pawlikowska-Pawlęga B., Dziubińska H., Król E. (2014). Characteristics of quercetin interactions with liposomal and vacuolar membranes. *Biochimica et Biophysica Acta*.

[23] Giakoumis E. G. (2018). Analysis of 22 vegetable oils’ physico-chemical properties and fatty acid composition on a statistical basis, and correlation with the degree of unsaturation. *Renewable Energy*.

[24] Nanlohy H. Y., Wardana I. N. G., Hamidi N., Yuliati L., Ueda T. (2018). The e ff ect of Rh^3+^ catalyst on the combustion characteristics of crude vegetable oil droplets. *Fuel*.

[25] Marlina E., Wijayanti W., Yuliati L., Wardana I. N. G. (2020). The role of pole and molecular geometry of fatty acids in vegetable oils droplet on ignition and boiling characteristics. *Renew Energy*.

[26] Fang H., Ni K., Wu J., Li J., Huang L., Reible D. (2019). The effects of hydrogen bonding on the shear viscosity of liquid. *Water*.

